# Trends, Implications, and Outcomes in the Changing Landscape of Cardiac Surgery: Transcatheter Aortic Valve Implantation in Patients with Severe Aortic Stenosis and Concomitant Coronary Artery Disease

**DOI:** 10.21470/1678-9741-2024-0050

**Published:** 2025-02-11

**Authors:** Kyriakos Spiliopoulos, Dimitrios Magouliotis, Thanos Athanasiou, John Skoularigis, Andrew V. Xanthopoulos

**Affiliations:** 1 Department of Cardiothoracic Surgery, University of Thessaly, Larissa, Greece. E-mail: kyriakosspili@aol.com; 1 Department of Cardiothoracic Surgery, University of Thessaly, Larissa, Greece; 1 Department of Cardiothoracic Surgery, University of Thessaly, Larissa, Greece; 2 Department of Cardiology, University of Thessaly, Larissa, Greece; 2 Department of Cardiology, University of Thessaly, Larissa, Greece

Calcified aortic valve stenosis, the most common acquired valvular pathology, often occurs in patients of advanced age, who are also at higher risk for atherosclerotic disease. As the population ages, the incidence of aortic valve pathology with concomitant coronary artery disease (CAD) is increasing. In the field of cardiac surgery, the approach of combined coronary artery bypass grafting (CABG) and aortic valve replacement (AVR) represents currently the third most frequently performed procedure behind isolated CABG and AVR^[1]^. However, the evolution of transcatheter aortic valve implantation (TAVI) into a first-choice treatment for high-risk and elderly patients delivers an alternative in the arsenal of treatment options, involving less invasive methods like TAVI + percutaneous coronary intervention (PCI) beside the conventional surgical approach of AVR + CABG. In this context, we read with great interest the article recently published by this journal entitled “Five-Year Follow-Up After Transcatheter Aortic Valve Implantation in Patients with Severe Aortic Stenosis and Concomitant Coronary Artery Disease: A Single-Center Experience”, by Abawi A et al.^[2]^, which evaluated the impact of CAD in patients undergoing TAVI. The issue is very relevant, and we would like to take the chance to add some comments about the management of this entity.

Although the impact of concurrent CABG procedure at the time of AVR remains controversial, several studies showed that it was not associated with increased mortality^[3]^. The working hypothesis explaining this observation is that surgical revascularization firstly neutralizes the adverse effects of CAD, and secondly, improves myocardial metabolism and reduces the risk of ischemia in hypertrophied left ventricles^[4]^. However, in the study of Abawi A et al.^[2]^, there are several missing data regarding the coronary revascularization, including its extent (complete *vs.* incomplete), type of grafting in case of CABG (arterial *vs.* vein; total arterial revascularization or not), the severity of the CAD (one vessel *vs.* multivessel disease, significant left main stem stenosis), as well as the time frame between the coronary revascularization and the TAVI procedure. All this information may reveal the precise CAD burden and its influence on the patients’ prognosis. Strictly speaking, according to the flow chart of the series, the real impact of the disease is measurable only in the 25 so called “non-intervened” CAD cases, as long as the rest involves, generally described, treated, “healed”(?) CAD.

Noteworthy, looking at the current literature dealing with the issue, the significance of CAD and severity of coronary lesions are differently defined among researchers arising from the lack of complete objective hemodynamic assessment using beside the golden standard of coronary angiography, diagnostic tools like fractional flow reserve (FFR), instantaneous wave-free ratio, and non-invasive methods, such as computed tomography angiography (CTA) and FFR-CTA. Furthermore, other sources of bias may be the variability between composite clinical endpoints, as well the limited follow-up^[5]^.

Nevertheless, the superiority in terms of outcomes of less invasive methods like TAVI + PCI over the conventional AVR + CABG has yet to be proven. In the current literature, only a few series addressed the issue. The most prominent of those, the recently published prospective randomized SURTAVI trial of intermediate risk patients after either TAVI + PCI or AVR + CABG presented favorable and comparable outcomes regarding all-cause mortality and disabling stroke at two years of follow-up with 16% and 14% (*P*=0.69), respectively^[6]^. While Abawi et al.^[2]^ in their study conclude that “five-year mortality did not differ between TAVI patients with or without CAD”, there are other reports where isolated TAVI performs better in patients without, compared to those with previous CABG-surgery^[7]^, or is superior compared to cases after TAVI + PCI^[8]^, indicating that there is still a significant lack of convincing evidence.

Another subject of debate in the topic remains the timing of the interventional strategies. All approaches (revascularization/PCI before, simultaneously, or after TAVI) have their advantages and drawbacks and are controversially discussed. Worth mentioning, the combined approach of concomitant PCI/revascularization and TAVI procedure is associated with a higher incidence of acute kidney injury due to increased volume of contrast infusion. Therefore, if a staged procedure is chosen, it is recommended to keep a period of at least three weeks between both interventions^[3]^. Conclusively, the decision for the therapeutical strategy, involving several disciplines as a Heart Team, should be individualized based upon the patient’s profile, characteristics, symptoms, and preferences, as well as the anatomical feasibility of the procedure^[5]^.

In general, the employment of the TAVI technique in revascularized CAD patients enables favorable outcomes, especially in certain subsets of high-risk cases that are unable to undergo conventional heart surgery. Nevertheless, this approach has yet to be further evaluated through large prospective randomized trials with midand long-term follow-ups.

## References

[1] Kobayashi KJ, Williams JA, Nwakanma LU, Weiss ES, Gott VL, Baumgartner WA (2009). EuroSCORE predicts shortand mid-term mortality in combined aortic
valve replacement and coronary artery bypass patients. J Card Surg.

[2] Abawi A, Magnuson A, Fröbert O, Samano N. (2023). Five-year follow-up after transcatheter aortic valve implantation
in patients with severe aortic stenosis and concomitant coronary artery
disease: a single-center experience. Braz J Cardiovasc Surg.

[3] Spiliopoulos K, Magouliotis D, Angelis I, Skoularigis J, Kemkes BM, Salemis NS (2023). Concomitant valve replacement and coronary artery bypass grafting
surgery: lessons from the past, guidance for the future? A mortality
analysis in 294 patients. J Clin Med.

[4] Formica F, Mariani S, D'Alessandro S, Singh G, Di Mauro M, Cerrito MG (2020). Does additional coronary artery bypass grafting to aortic valve
replacement in elderly patients affect the early and long-term
outcome?. Heart Vessels.

[5] de Azevedo Filho AF, Accorsi TA, Ribeiro HB. (2021). Coronary artery disease in patients with aortic stenosis and
transcatheter aortic valve implantation: implications for
management. Eur Cardiol.

[6] Søndergaard L, Popma JJ, Reardon MJ, Van Mieghem NM, Deeb GM, Kodali S (2019). Comparison of a complete percutaneous versus surgical approach to
aortic valve replacement and revascularization in patients at intermediate
surgical risk: results from the randomized SURTAVI trial. Circulation.

[7] Kawashima H, Watanabe Y, Kozuma K, Kataoka A, Nakashima M, Hioki H (2018). Comparison of midterm outcomes of transcatheter aortic valve
implantation in patients with and without previous coronary artery bypass
grafting. Heart Vessels.

[8] Kotronias RA, Kwok CS, George S, Capodanno D, Ludman PF, Townend JN (2017). Transcatheter aortic valve implantation with or without
percutaneous coronary artery revascularization strategy: a systematic review
and meta-analysis. J Am Heart Assoc.

